# Prognostic predictors of lymph node metastasis in penile cancer: a systematic review

**DOI:** 10.1590/S1677-5538.IBJU.2020.0959

**Published:** 2021-01-20

**Authors:** David S. Zekan, Ahmad Dahman, Ali J. Hajiran, Adam M. Luchey, Jad Chahoud, Philippe E. Spiess

**Affiliations:** 1 West Virginia University Department of Urology MorgantownWV USA Department of Urology, West Virginia University, Morgantown, WV, USA.; 2 H. Lee Moffitt Cancer Center & Research Institute Department of Genitourinary Oncology TampaFL USA Department of Genitourinary Oncology, H. Lee Moffitt Cancer Center & Research Institute, Tampa, FL, USA.

**Keywords:** Penile Neoplasms, Lymphatic Metastasis, Health Belief Model

## Abstract

**Purpose:**

Squamous cell carcinoma (SCC) of the penis is a rare disease in developed countries but is associated with significant morbidity and mortality. A crucial prognostic factor is the presence of inguinal lymph node metastases (ILNM) at the time of diagnosis. At least 25% of cases have micrometastases at the time of diagnosis. Therefore, we performed a literature review of studies evaluating factors, both clinical and pathological, predictive of lymph node metastases in penile SCC.

**Materials and methods:**

Studies were identified using PubMed and search terms included the following: penile cancer, penile tumor, penile neoplasm, penile squamous cell carcinoma, inguinal lymph node metastasis, lymph node metastases, nodal metastasis, inguinal node metastasis, inguinal lymph node involvement, predictors, and predictive factor. The number of patients and predictive factors were identified for each study based on OR, HR, or RR in multivariate analyses, as well as their respective significance values. These were compiled to generate a single body of evidence supportive of factors predictive of ILNM in penile SCC.

**Results:**

We identified 31 studies, both original articles and meta-analyses, which identified factors predictive of metastases in penile SCC. The following clinical factors were predictive of ILNM in penile SCC: lymphovascular invasion (LVI), increased grade, increased stage (both clinical and pathological), infiltrative and reticular invasion, increased depth of invasion, perineural invasion, and younger patient age at diagnosis. Biochemically, overexpression of p53, SOD2, Ki-67, and ID1 were associated with spread of SCC to inguinal lymph nodes. Diffuse PD-L1 expression, increased SCC-Ag expression, increased NLR, and CRP >20 were also associated with increased ILNM.

**Conclusions:**

A multitude of factors are associated with metastasis of SCC of the penis to inguinal lymph nodes, which is associated with poor clinical outcomes. The above factors, most strongly LVI, grade, and node positivity, may be considered when constructing a nomogram to risk-stratify patients and determine eligibility for prophylactic inguinal lymphadenectomy.

## INTRODUCTION

Squamous cell carcinoma (SCC) of the penis is a rare yet distressing condition associated with significant morbidity and mortality. In developing countries, however, this rate remains higher at up to 4.4 per 100.000 men. This is commonly attributed to a lower rate of circumcision and poor hygiene. It is especially rare in developed countries; the incidence in the United States is 0.81 cases per 100.000 men (1). Inguinal lymph nodes are not only the first site of metastatic spread, but also a crucial prognostic factor associated with penile SCC (2). Therefore, an accurate algorithm for screening and predicting lymph node involvement is crucial to management.

The 25% likelihood of micrometastatic disease at time of presentation of penile SCC creates further management dilemmas (2). 2020 National Comprehensive Cancer Network (NCCN) guidelines for management of non-palpable inguinal lymph node penile cancer include surveillance if low risk (cTis, cTa, cT1a) and chest/abdomen/pelvic imaging followed by inguinal lymph node dissection or dynamic sentinel lymph node biopsy (DSLNB) if intermediate or high risk (cT1b, cT2 or higher) (3). European Association of Urology (EAU) concur that lymph node staging should be offered if lymph nodes are nonpalpable and the patient is intermediate or high risk (4).

Prophylactic inguinal lymphadenectomy, while providing the best survival in clinically node-negative patients, can be overtreatment in patients that do not have micrometastases due to the high morbidity associated with the surgery. Studies have shown up to a 25% complication rate with the procedure, including skin necrosis, wound infection, lymphedema, seroma, lymphocele, and deep vein thrombosis (5, 6). Factors associated with higher risk of inguinal lymph node metastasis (ILNM) include higher pathologic tumor stage, higher grade, vascular or lymphatic invasion, and specific histologic features. However, identifying reliable predictors of metastasis, specifically micrometastasis, is crucial in the management of penile cancer. Therefore, we conducted a systematic review evaluating recent literature to better understand predictors of penile SCC LNM.

## MATERIALS AND METHODS

This systematic literature review was conducted using studies performed between 2000 and 2020. Searches were conducted using PubMed and search terms included the following: penile cancer, penile tumor, penile neoplasm, penile squamous cell carcinoma, inguinal lymph node metastasis, lymph node metastases, nodal metastasis, inguinal node metastasis, inguinal lymph node involvement, predictors, and predictive factor. All studies pertinent to the topic were reviewed, and references meeting our inclusion criteria not generated by our PubMed search were manually extracted and reviewed as available.

Eligible studies for inclusion within this systematic review were selected based on the following: 1) precise definition of predictors; 2) sufficient sample size to generate statistically significant predictors of LNMs; 3) pathologically-confirmed LNMs; 4) English studies performed with human subjects; 5) Studies performed after 2000; 6) Studies analyzing SCC of the penis as opposed to other penile neoplasms.

Definitions of several predictors were defined as previously published (2). Clinically positive inguinal lymph nodes (cN+) were defined as those that are palpable or visible with imaging examinations. Histological grade was divided into three groups: G1 (well-differentiated), G2 (moderately differentiated), and G3 (poorly differentiated). TNM staging used was based on that defined by the NCCN penile cancer guidelines (3). Comparison of stages to reach statistically significance varied between studies (Table-1). Growth pattern was classified as superficial or vertical; Invasion depth was measured from the intact basement membrane at the edge of the primary tumor to the deepest infiltrating tumor cell. LVI was defined as the presence of cancer in the lymphatic or vascular lumen that was detected by immunohistochemical staining (2). Histopathological subtypes were classified as low risk (verrucous, papillary, and warty), intermediate risk (usual SCCs and mixed forms), and high risk (basaloid, sarcomatoid, adenosquamous, and poorly differentiated types) according to EAU guidelines (4). PD-L1, Ki-67, SOD1, and ID1 expression and P53 immunohistochemistry were measured in tumor. CRP, NLR, and SCC-Ag were measured in serum (2). Apparent diffusion capacity was obtained on diffusion-weighted MRI of the penis and pelvis (7).

**Table 1:** Original studies reviewed with factors examined for lymph node metastasis and corresponding statistical significance on multivariate analysis.

Original Studies						
Study	Patients (N)	Definition of LNM	LNM (n) (%)	Predictors of LNM	OR (95% CI)	p-value (multivariate)
Peak et. al. (2019) (1)	1636	NR	NR	Grade:		0.002
				G2 (vs. G1)	2.58 (1.39-4.79)	
				G3-4 (vs. G1)	3.27 (1.70-6.29)	
				LVI	2.49 (1.61-3.84)	<0.0001
				cN+:	20.0 (11.4-35.7)	<0.0001
				N1 vs. N0	27.8 (14.1-55.6)	
				N2 vs. N0	49.2 (14.8-162.8)	
				N3 vs. N0		
Qu et. al. (2018) (5)	380	≥N1	63 (17)	Age ≤60	0.68 (0.52-0.88)	0.003
				≥T1b	3.32 (1.38-8.01)	0.0075
				G2 (vs. 1)	2.98 (1.26-7.62)	0.023
				G3 (vs. 1)	3.97 (1.32-11.9)	0.014
				T2a	0.341 (0.111-1.049)	0.061
				T2b	2.20 (0.399-12.120)	0.365
				T3	0.075 (0.012-0.462)	0.005
Maciel et. al. (2019) (34)	65	≥N1	24 (37)	G2	0.731 (0.282-1.893)	0.518
				G3	1.489 (0.145-15.235)	0.737
				LVI	5.965 (0.857-41.507)	0.071
				P53 expression	1.789 (0.602-5.318)	0.296
				≥T2	NR	0.079
				≥G2	NR	0.118
				LVI	5.35 (1.009-28.313)	0.049
Zhu et. al. (2007) (19)	73	≥N1	30 (41)	High p53	6.01 (1.402-25.764)	0.016
				High Ki-67	NR	0.861
				High E-cadherin	NR	0.089
				High MMP-9	NR	0.852
				≥T2	NR	0.012
				Vascular invasion	NR	0.005
				50+% different	NR	0.043
Slaton et. al. (2001) (35)	48	≥N1	18 (38)	G2+	NR	0.393
				≥20 mitoses/10hpf	NR	0.196
				Tumor depth	NR	0.522
				Tumor thickness	NR	0.786
				Tumor thickness	0.78 (0.27-2.21)	0.6378
				Vertical growth pattern	2.40 (0.84-6.80)	0.1008
				G2-3	0.79 (0.28-2.25)	0.1110
				LVI	15.48 (5.37-44.61)	<0.0001
Ficarra et. al. (2006) (36)	175	N+	71 (41)	Corpora cavernosa infiltr	1.76 (0.69-4.53)	0.2387
				Corpus spongiosum infiltr	2.30 (0.87-6.05)	0.0915
				Urethra infiltr	1.55 (0.50-4.82)	0.4519
				cN+	6.14 (2.44-15.43)	0.0001
				LVI	6.75 (1.28-35.73)	0.024
				T2a	2.61 (0.68-10.1)	0.17
				T2b	7.32 (0.66-81.52)	0.10
Zhu et. al. (2010) (6)	110	≥N1	26 (24)	T3	3.78 (0.44-32.66)	0.22
				G2	2.77 (0.72-10.72)	0.14
				G3	6.89 (0.77-61.88)	0.09
				Strong p53	3.22 (0.96-10.86)	0.058
Velazquez et. al. (2008) (37)	134	N+	66 (49)	PNI	NR	0.001
				High grade	NR	0.0001
				High grade	14.68 (2.40-89.87)	0.004
Bhagat et. al. (2010) (38)	53	pN+	22 (42)	LVI	9.83 (1.71-56.57)	0.01
				cN+	7.78 (0.97-62.18)	0.05
				LVI	3.1 (1.4-6.9)	<0.05
				T2	1.50 (0.58-3.88)	>0.05
Winters et. al. (2016) (39)	206	pN1+	51 (25)	T3/4	1.52 (0.57-4.01)	>0.05
				G3/4	1.38 (0.66-2.88)	>0.05
				LVI	2.173 (1.094-4.320)	0.027
				Grade:		0.011
				Intermediate	3.309 (1.223-8.949)	
				Poor	4.874 (1.730-13.730)	
Graafland et. al. (2010) (40)	342	N+	68 (20)	Corpus spongiosum invasion	1.465 (0.738-2.909)	0.28
				Corpus cavernosum invasion	1.591 (0.782-3.234)	0.20
				Urethral invasion	0.906 (0.360-2.279)	0.83
				≥T1b	2.67 (1.16-6.15) *	0.02
Fonseca et. al. (2013) (8)	82	N+	46 (56)	LVI	2.09 (1.03-4.22) *	0.04
				Infiltrative invasion	2.00 (1.00-4.03) *	0.03
				T2-3 (vs. 1)	NR	0.004
Dai et. al. (2006) (41)	72	≥N1	23 (32)	G2/3	NR	0.207
				Tumor depth	NR	<0.001
				Tumor depth	NR	0.03
				Vascular invasion	NR	0.02
Age	NR	0.24
Emerson et. al. (2001) (42)	22	≥N1	10 (45)	Stage	NR	0.28
				Grade	NR	0.53
				Carcinoma in situ	NR	1.00
				cN+	8.9 (2.7-29.2)	<0.001
				PNI	9.6 (2.7-33.6)	<0.001
Termini et. al. (2015) (10)	125	N+	44 (35)	Tumor depth	11.6 (1.4-97.1)	0.023
				SOD2 overexpression	3.4 (1.1-10.1)	0.029
				LVI	7.224 (0.831-22.730)	0.029
				Absent koilocytosis	0.088 (2.628-50.718)	0.001
Nascimento et. al. (2020) (14)	55	pN+	28 (51)	Grade	2.333 (0.101-2.232)	0.288
				cN+	1.106 (0.023-0.821)	0.888
				PNI	0.24 (0.126-2.488)	0.099
				Stage	1.389 (0.124-2.017)	0.649
				G2 (vs. 1)	2.8 (0.997-7.459)	0.04
				G3 (vs. 1)	6.8 (2.560-19.793)	<0.001
				Stage:		0.362
				pT2	3.8 (0.836-16.406)	
Ramkumar et. al. (2009) (43)	200	pN1+	31 (16)	pT3-pT4	3.1 (0.725-26.361)	
				Extent of penile surgery:		0.49
				Partial	0.3 (0.208-4.798)	
				Total	0.3 (0.177-6.303)	
Warli et. al. (2020) (21)	48	N+	34 (71)	Ki-67	NR	0.045
				G2 (vs. 1)	26.52 (2.29-306.86)	0.0087
				G3 (vs. 1)	44.92 (3.34-604.66)	0.0041
Alkatout et. al. (2011) (9)	72	N+	34 (47)	cN+	3.30 (0.97-11.16)	0.0554
				Reticular invasion	5.64 (1.56-20.43)	0.0084
				cN+	8.58 (3.37-21.87) **	<0.001
				T2 (vs. 1)	6.37 (1.67-24.35) **	0.007
				T3-4 (vs. 1)	10.98 (1.59-75.64) **	0.015
				G2 (vs. 1)	7.62 (3.106-18.74) **	<0.001
Wang et. al. (2018) (44)	198	N+	96 (48)	G3-4 (vs. 1)	9.13 (2.00-41.57) **	0.004
				Intermediate risk histology	3.66 (1.30-10.37) **	0.021
				High risk histology	28.74 (2.37-348.54) **	0.008
				LVI	2.84 (0.40-20.01) **	0.296
				High grade	NR	0.02
				Lymphatic invasion	NR	0.02
Ficarra et. al. (2002) (45)	30	pN+	9 (30)	Vascular invasion	NR	0.97
				Corpora cavernosa invasion	NR	0.84
				Urethra infiltration	NR	0.77
				CRP >20	NR	0.04
				Residential area	NR	0.5
Al Ghazal et. al. (2013) (22)	51	N+	16 (31)	BMI	NR	0.9
				Age	NR	0.9
				Stage	NR	0.01
				Grade	NR	0.1
				G3-4 (vs. 1)	6.467 (1.241-33.684)	0.027
Zhou et. al. (2020) (46)	75	≥N1	31 (41)	LVI	5.162 (1.056-25.243)	0.043
				Short diameter to largest clinical LN	1.349 (1.133-1.606)	0.001
				G2 (vs. 1)	2.16	0.02
Unadkat et. al. (2020) (47)	590	pN+	142 (24)	G3-4 (vs. 1)	2.81	<0.001
				LVI	3.12	<0.001
				Diffuse PD-L1 expression	NR	<0.01
Ottenhoff et. al. (2017) (17)	213	N+	66 (31)	cN+	3.83 (1.4-10.0)*	<0.05
				Lymphatic invasion	3.95 (1.5-10.4) *	<0.05
Guimaraes et. al. (2006) (48)	112	N+	55 (49)	Infiltrating invasion	4.18 (1.5-11.3)*	0.005
				Radiograph LN	NR	0.001
Luchey et. al. (2014) (49)	51	pN+	31 (61)	Age <65	NR	0.049
Li et. al. (2019) (50)	891	N1-N3	166 (19)	LVI	NR	<0.001
Lopes et. al. (2002) (20)	82	N+	42 (51)	p53 overexpression	4.8 (1.6-14.9) *	<0.05
				Lymphatic embolization	9.4 (2.8-31.6) *	<0.05
Barua et. al. (2018) (7)	26	N+	NR	Apparent diffusion capacity on DW-MRI	NR	0.001
Hu et. al. (2019) (51)	64	N+	26 (41)	ID1 overexpression	NR	0.007

* RR;

** HR

**LVI** = lymphovascular invasion;

**PNI** = perineural invasion;

**cN+** = clinically node positive;

**pN+** = pathologically node positive;

**MMP-9** = matrix metalloprotease 9;

**SOD2** = superoxide dismutase 2;

**PD-L1**= programmed death-ligand 1;

**ID1** = DNA-binding protein inhibitor ID-1

Numbers of subjects (N) within individual original research articles were extracted as well as number of lymph node metastases (#LN; as available). Statistically significant and insignificant prognosticators (with p-values) were also collected.

## RESULTS

Original research articles analyzing clinical, histopathologic, and biochemical predictors of LNMs in SCC of the penis are presented in Table-1, including number of patients within the study (N), definition of positive lymph nodes, number of patients with LNMs (n), percentage of metastases within the study population (%), and factor(s) shown to be predictive of lymph node metastases within the study (with p-value). Statistically significant predictors present in greater than one study are shown in Table-2, with total number of patients and lymph nodes presented as available within the reviewed manuscripts.

**Table 2:** Quantity of clinical/pathological markers found to be significant amongst all studies.

Quantity of clinical/pathological markers:		
Predictor	Studies	Patients with ILNM/ Total patients (%)
LVI	17	815/2946 (28)
Grade	11	606/2074 (29)
cN+	8	295/611 (48)
Stage	6	270/845 (32)
Invasion pattern	3	148/266 (56)
Tumor depth	3	77/219 (35)
Age	2	94/431 (22)
PNI	2	110/259 (42)

### 

#### Clinical/Pathological Factors

Factors known to worsen prognosis for patients with SCC of the penis correlate strongly with positive lymph node(s) on inguinal lymphadenectomy. Namely, on our review, lymphovascular invasion was shown in both the highest number of studies and patients to correlate with lymph node metastases in patients with SCC of the penis. In their analysis of 1636 patients, all of whom had pathological lymph node staging, Peak et al. demonstrated lymphovascular invasion in 20.6% of patients with odds ratio (OR) of 2.49 (1). Similarly, higher grade and stage, as well as clinically positive nodes on exam were shown to be predictors of positive pathological involvement of lymph nodes. Specifically, 47.4% of patients were G2 and 31.7% G3-4, with respective ORs of 2.58 and 3.27. Both pathological and clinical staging were significant predictors of LNM in this study, with OR of 1.61 and 1.50 in p2 vs. p3/4 and 23.3, 43.5, and 76.0 in cN1, cN2, and cN3, respectively (1). Although less reported, infiltrative (RR=2.68; present in 70.2% with ILNM) and reticular invasion (present in 64% with ILNM) of the primary lesion on pathologic examination were also significant predictors of positive lymph nodes in SCC of the penis (8, 9). Finally, increased depth of invasion, perineural invasion, and decreased patient age at diagnosis were shown to have predictive value; 90% patients with tumor depth <=5mm had ILNM, while 48.8% >5mm had metastases. Similarly, 73.5% of patients with perineural invasion had ILNM compared to 24.4% without perineural invasion (10). Age varies amongst studies, but Qu et al. note the average age at diagnosis in patients with ILNM to be 62 compared to 69 in those without (5).

A common, and seemingly reasonable, method for determination of patients who should undergo a full inguinal lymph node dissection is through the use of dynamic sentinel lymph node biopsy (DSLNB). The NCCN and EAU both recommend use of DSLNB in patients with intermediate- and high-risk disease who have non-palpable inguinal nodes on clinical exam. Based on their literature review and nomogram, Peak et al. suggest that this should only be performed in centers specialized in lymph node mapping by clinicians who focus in penile cancer (1). This is due to a reported 6% false-negative rate reported by Lam et al. (11). Another group performing similar work using a large institutional database cited a 7% false-negative rate and noted the cost associated with DSLNB may outweigh the benefit of extended inguinal node dissection. Schubert et al. performed a smaller study (32 patients) with sentinel node sampling followed by inguinal node dissection in positive cases according to EAU guidelines and showed no false negatives (12). Underscored throughout are the risks associated with DSLNB, which are similar albeit less severe than those associated with a full inguinal dissection and occur at a rate of 7.6%: wound infection, lymphocele, and hematoma (11). Dell’Oglio et al. suggest that a combination radioactive (99mTc-nannocolloid) and fluorescent (indocyanine green) tracer can increase the sensitivity of DSLNB over regular a combination of radiotracer and blue dye. Specifically, in a cohort of 400 patients, they showed a 39% higher sentinel node detection rate, further increasing the sensitivity of this nodal detection measure and its clinical utility (13).

Interestingly, one study showed that absence of koilocytosis (seen in epithelial cells with HPV infection) was predictive of metastasis; specifically, 32.2% of patients with histological koilocytosis had positive nodes compared to 82% without koilocytosis (14). Also, a more easily obtained, but less studied factor that correlates with metastasis is the apparent diffusion coefficient (ADC) on diffusion-weighted MRI (DW-MRI) of the primary tumor, which shows the changes in proton mobility when there is underlying pathology or tissue alteration. ADC is lower in the setting of lymph node metastases, even when nodes are of normal size; one study yielded a sensitivity of 100% and positive predictive value of 84.61% (7). Other advances with MRI in the detection of ILNM in penile cancer involve the use of ultra-small superparamagnetic iron oxide particles (USPIO) as contrast agents. These agents are taken up by penile lymphatics and phagocytosed by resident macrophages; these macrophages are less prevalent in metastatic nodes. In a limited study with seven men (stage T1b-T2), this detection method showed sensitivity of 100%, specificity of 97%, positive predictive value of 81.2%, and negative predictive value of 100% (15, 16). This provides promise as PET/CT is only 57% accurate in predicting ILNM in patients with normal groin exams compared to 96% in patients with palpable nodes (17). Conventional imaging modalities rely on size criteria (>8-10mm) to diagnose ILNM. In patients who are low-risk for ILNM, an 8mm cut-off in the CT short axis provides the most accurate detection, with a sensitivity of 87% and a specificity of 81%. For patients with high risk for ILNM, size is less accurate, and the most accurate (88%) criteria for nodal involvement is an irregular nodal border with a specificity of 95% (15). Moving away from size criteria for the evaluation of ILNM in the presence of known primary SCC of the penis is crucial, as this has the tendency to miss occult metastases in normal-sized nodes and to label reactive nodes as malignant. This led Singh et al. to label overall cross sectional imaging (CT and MRI) detection of ILNM with a sensitivity of 40-60% and a false negative rate of 10-20%. However, these imaging methods are helpful in detecting metastases in the pelvis/retroperitoneum and in patients whose body habitus limit physical examination (18).

#### Biochemical Factors

Less studied predictors of LNM in SCC of the penis that remained statistically and clinically significant were noted in individual studies for the purposes of this review. The majority of these studies are biochemical markers shown to be under- or over-expressed in the tumor or blood of study subjects. Namely, tumor suppressor p53 overexpression was shown to predict migration of primary tumors to inguinal lymph nodes (19, 20). The antioxidant and tumor suppressor superoxide dismutase (SOD2) overexpression (overexpression=present in >50% of cells; seen in 44.8% of penile SCCs) was also predictive of lymph node involvement: 52.8% of patient with nodal involvement had the above criteria for overexpression compared to 24.6% with <50% of cells overexpressing (10). Warli et al. recently reported that overexpression (>20% of nuclei) of the nuclear proliferative protein Ki-67 is associated with increased movement of SCC of the penis to inguinal lymph nodes independent of tumor stage and grade (21). Diffuse PD-L1 expression is significantly predictive, which serves as a clinically relevant marker because of recent advancements targeting PD-L1 with immunotherapeutic agents (17). Tumor overexpression of ID1, which encodes a DNA-binding protein inhibitor (effectively eliminating its DNA-binding ability) is also known to predict node metastasis. Blood level of CRP >20mg/dL was the only predictive factor in the original research articles reviewed that could be detected in the serum (22).

#### Pertinent Meta-Analyses

Various other reviews have sought to define primary tumor characteristics predictive of lymph node metastasis in order to better define the need for prophylactic inguinal lymphadenectomy in SCC of the penis, many of which overlap with the above original studies. Namely, Ficarra et al. suggest histologic subtype, pathologic extension, histologic grade, and lymphatic and/or venous embolization are the most important factors (23). Specifically, basaloid SCC, >pT1, and >G1 predict higher risk of lymph node metastasis and poor prognosis. Lymphatic embolization is a pathologic diagnosis with nests of carcinomatous cells in a lumen with thin walls, without smooth muscle fibers or red blood cells. The same condition with red blood cells or smooth muscle fibers is considered venous embolization, both of which suggest the need for inguinal lymphadenectomy (24). Hu et al. performed a meta-analysis of retrospective studies and showed both clinicopathologic and biochemical markers to be associated with increased risk of inguinal LNM (2). In addition to the clinicopathologic factors cited by Ficarra et al., they showed positive clinical nodes, vertical growth, tumor size (>3cm), invasion depth (>5mm), and nerve, corporal, and urethral invasion to be predictors of lymph node metastasis. They also added higher neutrophil-to-lymphocyte ratios (NLR) and squamous cell carcinoma antigen (SCC-Ag) overexpression to the above list of biochemical predictors (23). Zhou et al. performed a meta-analysis of exclusively perineural invasion and its ability to predict inguinal lymph node metastasis; they showed a statistically significant higher rate of LNM in penile SCC with perineural invasion compared to that in which nerve invasion is absent (25).

## DISCUSSION

Development of an algorithm capable of accurately predicting ILNM in patients with SCC of the penis is crucial, as adequate lymph node dissection has been established to improve survival in these patients for almost forty years (26). Most of the above clinicopathologic factors associated with increased risk of SCC of the penis metastasis to inguinal lymph nodes are intuitive and already established as factors making the disease intermediate- or high-risk according to the NCCN. In patients with non-palpable inguinal lymph nodes, this includes T1b disease and any disease T2 or higher (3). As above, these patients are candidates for DSLNB per the NCCN and EUA. In patients with palpable inguinal nodes, the NCCN suggests movement straight to ILND if the lesion is high risk: T1, high-grade, lymphovascular invasion, perineural invasion, or >50% poorly differentiated. Percutaneous biopsy is only suggested in patients with low risk disease (3). Essentially, our review concurs with and further compliments the NCCN guidelines with addition of the following clinically- and pathologically significant factors: decreased patient age at diagnosis, absence of koilocytosis, and decreased apparent diffusion coefficient on DW-MRI. Although further cost analyses need to be performed for the latter, age and koilocytosis on pathological section provide easily obtained measures to increase clinical suspicion of ILNM in patients with diagnosed SCC of the penis. With regard to imaging, EAU guidelines state that longitudinal/transverse diameter ratio and absence of the lymph node hilum are highly specific findings on ultrasound, CT/MRI cannot reliably detect micrometastasis, and PET/CT will not detect lymph node spread <10mm (4). However, our review suggests that use of novel MRI contrast agents can be helpful in the detection of ILNM in SCC of the penis.

More novel elements predicting the metastasis of penile SCC to inguinal lymph nodes are the biochemical factors in the form of tumor markers and serum tests outlined above. Certainly, inflammation plays some role in both the initiation and movement of primary penile SCC tumors to lymph nodes, as Hu et al. conclusively identified NLR, CRP, and PD-L1 as predictors of LNM (2). Neutrophilia and lymphopenia represent a systemic inflammatory response and an active immune response. Increased NLR has been shown to predict poor prognosis in castration-resistant prostate cancer, cervical adenocarcinoma, lung cancer, and esophageal carcinoma and is known to be an independent predictor of overall survival in SCC of the penis (2, 27). Similarly, CRP levels have been shown to predict poor prognosis in penile SCC patients, but mixed evidence exists for their ability to predict specifically ILNM (22, 28, 29). The transmembrane protein PD-L1 is important in the prognosis of penile SCC because of its ability to suppress the host immune system. High expression of this gene is related to increased LNM and poor prognosis, but it also serves as a common target for immunotherapy, reinforcing its theoretical benefit in penile cancer (2). Su et al. describe a case of metastatic recurrent SCC of the penis with PD-L1 expression >10% with positive response to immunotherapy with Toripalimab. Effective immunotherapy is crucial as 62% of patients are PD-L1 overexpressers, which is associated with metastasis and poor clinical outcome (30). SCC-Ag is another marker better-studied in SCC of the cervix, with varying individual results for prediction of LNM vs. solely tumor burden in SCC of the penis (31–33). However, Hu et al. conclusively showed with meta-analysis of available evidence that its elevation serves as a predictor of LNM in SCC of the penis (2). Markers that have been studied on a very limited basis (single studies) include ID1 and SOD2, both of which clearly warrant further research before their differential expression can definitively be called predictive of LNMs. However, the establishment of biomarkers as both predictors of metastases and therapeutic targets is crucial, as these tumor and serum markers are fairly easily obtained in addition to current staining, and can provide prognostic value guiding therapy as well as immunotherapeutic targets.

Obvious limitations with this review include a wide variation in the methods and included patient populations of original articles and systematic reviews/meta-analyses analyzed. This complicates performing another meta-analysis using this data. Similarly, our desire to outline a host of factors (both clinicopathological and biochemical) contributing to increased risk of LNM limits our ability to perform wider data analyses. Regardless, our collection of large patient populations through review of original research/meta-analyses generates risk factors that confidently predict LNM and allow for higher clinical suspicion and more aggressive management. Limited evidence for some factors, particularly age and biochemical predictors of LNM, makes it difficult to evaluate their clinical utility at present, and further work is necessary prior to their incorporation into guidelines.

## CONCLUSION

Here, we present a thorough review of available articles highlighting both clinicopathologic and biochemical factors predictive of LNM in patients with penile SCC. Although a specific nomogram is not presented, support is garnered for clinicians using clinically more aggressive grade and stage of tumors, as well as incorporation of imaging features and age of the patient, into risk stratification and decisions to sample nodes. Further, we present evidence for the use of inflammatory markers (CRP, NLR, PD-L1) and other tumor markers (p53, SCC-Ag, SOD2 and ID1 expression) in risk stratification. Clearly, a combination of these markers and clinical/pathological findings should be used as part of the shared decision-making model with patients suffering from SCC of the penis with potential LNM. Perhaps patients in whom clinical suspicion is high for ILNM would benefit from workup including the above blood and tumor markers as well as advanced imaging at the time of initial biopsy to support or counter the decision to perform ILND at the time of penectomy.
